# *N*-Substituted 5-Amino-6-methylpyrazine-2,3-dicarbonitriles: Microwave-Assisted Synthesis and Biological Properties

**DOI:** 10.3390/molecules19010651

**Published:** 2014-01-07

**Authors:** Ondrej Jandourek, Martin Dolezal, Pavla Paterova, Vladimir Kubicek, Matus Pesko, Jiri Kunes, Aidan Coffey, Jiahui Guo, Katarina Kralova

**Affiliations:** 1 Department of Medicinal Chemistry and Drug Analysis, Faculty of Pharmacy, Charles University in Prague, Heyrovskeho 1203, Hradec Kralove 50005, Czech Republic; E-Mail: dolezalm@faf.cuni.cz; 2 Department of Biological and Medical Sciences, Faculty of Pharmacy, Charles University in Prague, Heyrovskeho 1203, Hradec Kralove 50005, Czech Republic; E-Mail: pavla.paterova@fnhk.cz; 3 Department of Biophysics and Physical Chemistry, Faculty of Pharmacy, Charles University in Prague, Heyrovskeho 1203, Hradec Kralove 50005, Czech Republic; E-Mail: kubicek@faf.cuni.cz; 4 Department of Environmental Ecology, Faculty of Natural Sciences, Comenius University, Mlynska Dolina CH-2, Bratislava 842 15, Slovakia; E-Mail: matus.pesko@gmail.com; 5 Department of Inorganic and Organic Chemistry, Faculty of Pharmacy, Charles University in Prague, Heyrovskeho 1203, Hradec Kralove 50005, Czech Republic; E-Mail: kunes@faf.cuni.cz; 6 Department of Biological Sciences, Cork Institute of Technology, Bishopstown, Cork, Ireland; E-Mails: aidan.coffey@cit.ie (A.C.); gjh0517@yahoo.com (J.G.); 7 Institute of Chemistry, Faculty of Natural Sciences, Comenius University, Mlynska Dolina CH-2, Bratislava 842 15, Slovakia; E-Mail: kata.kralova@gmail.com

**Keywords:** pyrazinamide derivatives, *in vitro* biological evaluation, microwave-assisted synthesis, tuberculosis, lipophilicity

## Abstract

In this work a series of 15 *N*-benzylamine substituted 5-amino-6-methyl-pyrazine-2,3-dicarbonitriles was prepared by the aminodehalogenation reactions using microwave assisted synthesis with experimentally set and proven conditions. This approach for the aminodehalogenation reaction was chosen due to its higher yields and shorter reaction times. The products of this reaction were characterized by IR, NMR and other analytical data. The compounds were evaluated for their antibacterial, antifungal and herbicidal activity. Compounds **3** (R = 3,4-Cl), **9** (R = 2-Cl) and **11** (R = 4-CF_3_) showed good antimycobacterial activity against *Mycobacterium tuberculosis* (MIC = 6.25 µg/mL). It was found that the lipophilicity is important for antimycobacterial activity and the best substitution on the benzyl moiety of the compounds is a halogen or trifluoromethyl group according to Craig’s plot. The activities against bacteria or fungi were insignificant. The presented compounds also inhibited photosynthetic electron transport in spinach chloroplasts and the IC_50_ values of the active compounds varied in the range from 16.4 to 487.0 µmol/L. The most active substances were **2** (R = 3-CF_3_), **3** (R = 3,4-Cl) and **11** (R = 4-CF_3_). A linear dependence between lipophilicity and herbicidal activity was observed.

## 1. Introduction

*Mycobacterium tuberculosis* is counted with justification among the most dangerous and successful microorganisms in today’s world, especially in developing countries which have become the reservoir of resistant strains (the most burdened countries are in South Africa and East Asia) [1]. These strains are causing the biggest number of problems connected to tuberculosis (TB) treatment [2]. The main problem is resistance. It can be divided into three groups. The first one is called multidrug-resistant TB (MDR-TB) and these microbes are resistant to all first-line antituberculotic drugs (pyrazinamide - PZA, isoniazid - INH, rifampicin - RIF, ethambutol - ETH, streptomycin - STR). The second group is more treacherous. This type of resistance is called extensively or extremely drug-resistant TB (XDR-TB) with the resistance to the first-line anti-TB agents isoniazid and rifampicin together with the resistance to any fluoroquinolone used in the therapy and to at least one of three injectable second-line antituberculotic drugs (amikacin, kanamycin or capreomycin) [3]. The last and the latest category was the most dreaded and was called totally drug-resistant TB (TDR-TB) and the first case was recorded in India [4]. These mycobacterial strains were resistant to all current known therapy. Nowadays, this group has disappeared with the approval of bedaquiline for therapy of resistant forms of tuberculosis. Another problem is connected with the HIV pandemic. These two infectious diseases are influencing each other in a synergic way so this has resulted in efforts to develop new anti-tubercular agents.

This work deals with a microwave-assisted synthesis of pyrazinamide analogues with potential antimycobacterial activity. It is caused by the fact that pyrazinamide is counted among the first-line anti-tuberculosis drugs used in current therapy. Its unique ability to kill the dormant forms of *Mycobacterium tuberculosis* is crucial in shortening the time needed for the treatment, so PZA has sterilizing activity especially in combination with rifampicin [5].

PZA itself has multiple mechanisms of action. The first described was the activation of this prodrug *via* the enzyme pyrazinamidase (EC 3.5.1.19) to form pyrazinoic acid (POA). This metabolite causes a lowering of the inner compartment pH in mycobacterial cells. This leads to inhibition of membrane transport and then to cellular death [6,7,8]. The gene encoding this enzyme is called *pncA* gene and its mutation is responsible for the origin of mycobacterial resistance to PZA [9].

The second mechanism of action is connected with fatty acid synthase I (FAS I) (EC 2.3.1.85). It is suggested that the disruption of metabolism can be caused by inhibition of the cell membrane synthesis, which is essential for the survival of *Mycobacteria*, but this mechanism was mainly rejected for PZA itself by Boshoff because there is only low inhibition [10]. On the other hand, the PZA analogues such as 5‑chloropyrazinamide, esters of pyrazinoic acid and esters of 5‑chloropyrazinoic acid were proven to act in this way [11,12,13].

Recent research has suggested a novel mechanism of action of PZA - inhibition of *trans*-translation. This process is vital for survival and virulence of *Mycobacteria* and its inhibition leads to blockage of the proteosynthetic apparatus in ribosomes and to cellular death. These assumptions were proven by Zhang *et al.* [14].

Although PZA is the first-line antituberculotic drug, it was found that this molecule has exhibited other interesting biological activities such as antifungal, antibacterial, antiviral and antineoplastic effects [15,16,17,18,19,20].

There is another application of PZA derivatives that can be used in agriculture. The most successful pyrazine derivative diquat-dibromide (6,7-dihydrodipyrido[1,2-a:2',1'-c]pyrazinediium-dibromide), a non-selective, contact herbicide, which has been used to control many submerged and floating aquatic macrophytes, was found to interfere with the photosynthetic process by releasing strong oxidizers that rapidly disrupt and inactivate cells and cellular functions (at present banned in many EU countries) [21]. Many structural variations of pyrazine compounds with herbicidal properties can be found in the patent literature [22,23,24,25]. However, several pyrazine derivatives were also described as inhibitors of Hill reaction which inhibit photosynthetic electron transport (PET) in photosystem (PS) 2 [18,26]. The site of action of these PET inhibitors in the photosynthetic apparatus was situated predominantly on the donor side of PS2, in the section between oxygen evolving complex and intermediate D^·^, *i.e.*, tyrosine radical (Tyr_D_•) occurring on the 161^st^ position in D_2_ protein. Consequently, these compounds can be considered as PS2 herbicides which could have ultimately adverse effect on plant growth. In general, the PET-inhibiting effectiveness of pyrazine derivatives depends on compound lipophilicity and σ Hammett constants of individual substituents. Hosseini *et al.* studied the electronic and structural descriptors, which are the main factors for the cytotoxicity in the series of substituted *N*-phenylpyrazine-2-carboxamides [27].

This study is focused on preparation of *N*-substituted structural and functional derivative of PZA (5-chloro-6-methylpyrazine-2,3-dicarbonitrile) that was treated with ring-substituted benzylamines using the advantages of a microwave reactor. It should be stressed that this type of syntheses has become popular due to its higher yields, shorter reaction times or solvent savings in comparison with conventional organic syntheses [28]. One of the main advantages is the heating. It is uniform through the volume of the sample and the microwaves usually interact with molecules themselves not vessel sides. Another benefit is connected with the temperature reached by the solvent used. The final temperature is usually far higher than the standard boiling point of the solvent when using over- pressurized systems. It is reached and bypassed in seconds. Improved heating usually leads to higher yields and shorter reaction times. There is one limitation for choosing the conditions. It is the polarity of the solvent when the non-polar solvents cannot be used in the way the polar ones can be. If the polar solvent is used in the reaction, there is a direct coupling of microwaves with molecules. More polar solvents have greater ability to interact with microwave radiation. Using the solvents with low polarity (low absorbers) leads to longer times of heating and reaction. On the contrary, if the reagents themselves are polar it could lower the disadvantages of non-polar solvents. Finally there are new approaches to microwave accelerated methods using ionic liquids or solid phase reactions (adsorption on mineral oxides, phase transfer catalysis, neat reactions) [29]. Microwave assisted condensation in polar solvent is used in this work to accelerate the aminodehalogenation reaction. The conditions for the synthesis were proven experimentally. Antimycobacterial activity of the all prepared compounds was determined and compounds were evaluated also in relation to inhibition of photosynthetic electron transport (PET) in spinach (*Spinacia oleracea* L.) chloroplasts. The structure-activity relationships between the chemical structure and *in vitro* biological activities of evaluated compounds are discussed.

## 2. Results and Discussion

### 2.1. Chemistry

**Scheme 1:**
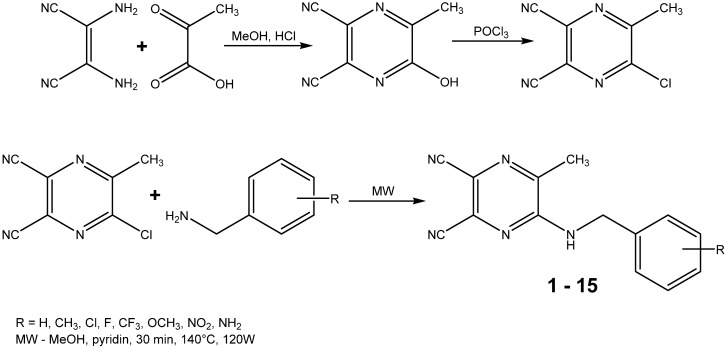
Synthesis of starting compound [30] and microwave assisted aminodehalogenation reaction resulting in a series of compounds **1**–**15**.

The starting compound 5-chloro-6-methylpyrazine-2,3-dicarbonitrile and the final compounds **1**–**15** were synthesized according to the general procedure shown in Scheme 1. The aminodehalogenation reaction of this starting compound and ring-substituted benzylamines yielded a series of 15 secondary amines of which 14 were novel. 5-(Benzylamino)-6-methylpyrazine-2,3-dicarbonitrile (**6**) was previously synthesised by Takematsu *et al.* and the reported melting point was 118–119 °C [30]. The compound we obtained melted at 128.7–130.7 °C. This difference can be caused by the mode of crystallization. All reactions were done using microwave reactor with focused field and yields were in the range between 17% and 62%. Lower yields were caused by the purification using preparative chromatography and recrystallization. It is also known that 3-nitro substitution, for which the yield was the lowest, is counted among the electron-withdrawing groups reducing the basicity of the amine nitrogen. Obtained analytical data were fully consistent with the proposed structures. Table 1 shows the substituents and other data of the synthesized compounds.

**Table 1:** Experimentally determined values of lipophilicity log *k*, calculated values of log *P*, electronic Hammett’s σ parameters and π parameters, 50% inhibition concentration IC_50_ [μmol/L] values related to PET inhibition in spinach chloroplasts in comparison with 3-(3,4-dichlorophenyl)-1,1-dimethylurea (DCMU) standard and *in vitro* antimycobacterial activity against *M. tuberculosis* H37Rv (minimal inhibition concentration (MIC) [μg/mL]) of compounds **1**–**15** compared to pyrazinamide (PZA) and isoniazid (INH) standards.

Compound	R	log *P*	log *k*	σ	π	IC_50_ [μmol/L]	MIC *M.tuberculosis* H37Rv [μg/mL]
1	2-CH_3_	3.41	0.4668	−0.17	0.1674	114.0	>100
2	3-CF_3_	3.84	0.5369	0.43	0.2375	37.7	12.5
3	3,4-Cl	4.04	0.7538	0.60	0.4544	16.4	6.25
4	4-CH_3_	3.41	0.5157	−0.17	0.2163	104.7	25
5	4-OCH_3_	2.8	0.2820	−0.27	−0.0174	464.6	>100
6	H	2.92	0.2994	0	0	-	25
7	4-NH_2_	2.12	−0.2014	−0.15	−0.5008	-	25
8	3-Cl	3.48	0.5172	0.37	0.2178	57.4	12.5
9	2-Cl	3.48	0.4663	0.22	0.1669	79.0	6.25
10	2-F	3.08	0.3042	0.06	0.0048	195.6	12.5
11	4-CF_3_	3.84	0.5626	0.51	0.2632	39.6	6.25
12	2-CF_3_	3.84	0.4865	0.51	0.1871	71.6	12.5
13	2,4-OCH_3_	2.67	0.3699	−0.55	0.0705	-	25
14	3-NO_2_	2.71	0.1808	0.71	−0.1186	487.4	12.5
15	4-Cl	3.48	0.5384	0.23	0.2390	86.5	12.5
PZA						-	12.5
INH						-	1.5625
DCMU						1.9	-

π = log *k*_(substituted)_ – log *k*_(unsubstituted)._

### 2.2. Calculated and Experimentally Set Lipophilicity

Lipophilicity is one of the most important factors that can affect the biological effect of the compound. It is connected with the membrane transport and other biological processes and it is also connected with solubility in the media. This physical-chemical property can be set experimentally. In this work we have used the Reversed Phase - High Performance Liquid Chromatography (RP-HPLC) methodology for measuring the capacity factors *k* with the calculation of log *k* that can be correlated to calculated values of lipophilicity log *P* (resp. Clog *P*). These calculations were carried out by using PC program CS ChemBioDraw Ultra 13.0. The results of these measurements are shown in the Table 1.

The lowest lipophilicity was shown by compound **7** (R = 4-NH_2_) and on the contrary, compound **3** (R = 3,4-Cl) was the most lipophilic compound of this series. Lipophilicity, based on log *k* values, increased for substituents in the benzyl part of the molecule as follows: 4-NH_2_ < 3-NO_2_ < 4-OCH_3_ < H < 2-F < 2,4-OCH_3_ < 2-Cl < 2-CH_3_ < 2-CF_3_ < 4-CH_3_ < 3-Cl < 3-CF_3_ < 4-Cl < 4-CF_3_ < 3,4-Cl 

**Figure 1:**
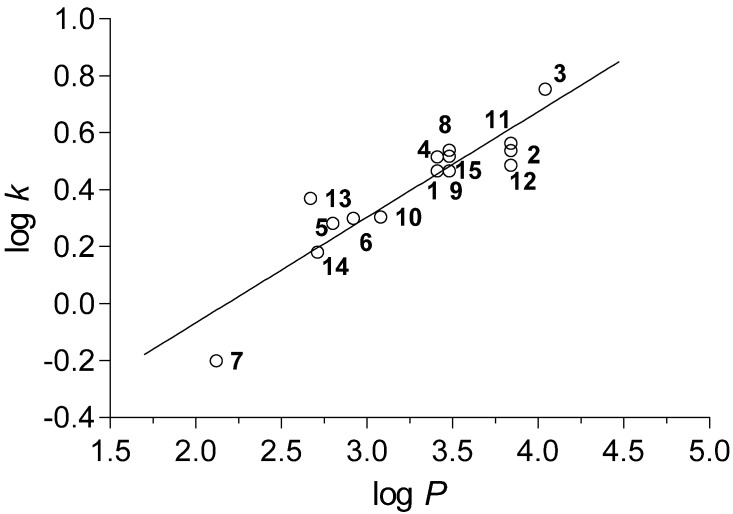
Plot of experimentally measured log *k* parameter on calculated log *P* (CS ChemBioDraw Ultra version 13.0).

The dependence of the measured log *k* parameters on the calculated log *P* values showed an approximate linearity, which is shown in Figure 1, and the corresponding correlation can be expressed by the following regression equation:

log *k* = −0.810 × (±0.150) + 0.371 × (±0.045) × log *P* r = 0.915, s = 0.092, F = 66.9, n = 15
(1)

### 2.3. Biological Assays

#### 2.3.1. Antimycobacterial *In Vitro* Screening

All prepared compounds were tested against four strains of *Mycobacterium*. These were *M. tuberculosis* and three non-tuberculosis strains. The most active substances against *M. tuberculosis* were compounds **3**, **9** and **11**. Their activity expressed as minimal inhibition concentration (MIC) was 6.25 µg/mL and the activities of standards were 12.5 µg/mL for PZA and 1.5625 µg/mL for INH. Nearly the whole series showed activity against *M. tuberculosis* H37Rv, which was in the range from 25 to 6.25 µg/mL, but compounds **11** and **12** were also active against other strains and both of them were active against *M. avium* 152. These strains are usually resistant or unsusceptible to pyrazinamide. This is the reason why INH was chosen as second standard. Obtained results can be compared with other synthesised compounds and a comparison can be drawn between more mycobacterial strains not only *M. tuberculosis*.

**Figure 2:**
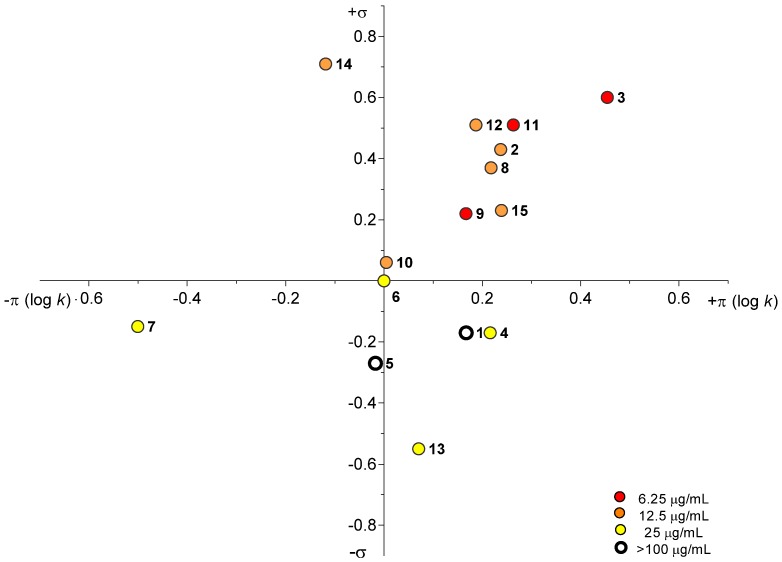
Dependence of antimycobacterial activity of studied compounds on the π constant (log *k*) as well as on the σ constant of R substituent.

As shown in Figure 2, dependence of antimycobacterial activity on lipophilicity expressed by π constant (log *k*) as well as on the σ constant of the R substituent was observed. The most active compounds (MIC = 6.25 µg/mL) were **3** (R = 3,4-Cl), **9** (R = 2-Cl) and **11** (R = 4-CF_3_) and were mentioned above. All of these compounds are situated in the right upper quadrant of the Craig’s plot. Further groups are represented by compounds **2** (R = 3-CF_3_), **8** (R = 3-Cl), **10** (R = 2-F), **12** (R = 2-CF_3_), **14** (R = 3-NO_2_) and **15** (R = 4-Cl) showing moderate activity with MIC = 12.5 µg/mL and compounds **4** (R = 4-CH_3_), **6** (R = H), **7** (R = 4-NH_2_) and **13** (R = 2,4-OCH_3_) with MIC = 25 µg/mL. Low antimycobacterial activity with MIC >100 µg/mL was exhibited by compounds **1** (R = 2-CH_3_) and **5** (R = 4‑OCH_3_). It can be stated that the activity is more dependent on π constant than on σ constant. On the other hand this dependence is not unambiguous. However, it is clear that the lipophilicity is important for antimycobacterial activity and the most suitable ring substituents are electron-withdrawing groups such as halogen or trifluoromethyl moieties.

#### 2.3.2. Antimycobacterial *In Vitro* Screening Against *M. smegmatis*

This evaluation was performed against fast-growing *Mycobacterium smegmatis* using isoniazid and ciprofloxacin as standards. None of the compounds showed activity against this mycobacterial strain. This may be caused by the fact that this fast-growing mycobacterium is less susceptible to antibiotic treatment.

#### 2.3.3. Antifungal and Antibacterial *In Vitro* Screening

This evaluation was performed in order to obtain results for antifungal and antibacterial activity against eight fungal strains and eight bacterial strains. None of the prepared compounds showed antibacterial activity against the tested strains. On the contrary, compounds **7** (MIC = 500 µmol/L), **10** (MIC = 500 µmol/L) and **12** (MIC = 125 µmol/L) exhibited activity against *Trichophyton mentagrophytes* but in comparison to standards, this activity was negligible. It can be also caused by the bigger susceptibility of this fungal strain being evaluated.

#### 2.3.4. Herbicidal Activity of Prepared Compounds

**Figure 3:**
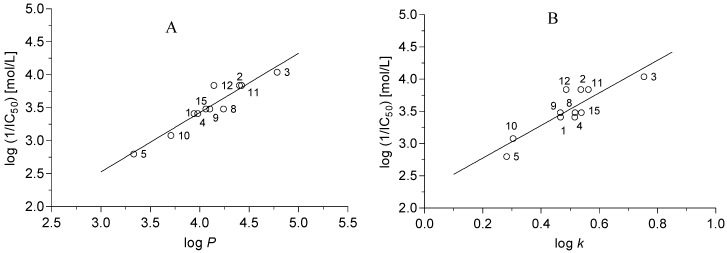
Dependence of PET inhibiting activity on the log *P* (**A**) or log *k* (**B**) of compounds **1**–**15**.

The studied compounds inhibited photosynthetic electron transport in spinach chloroplasts (Table 1). The PET inhibiting activity of studied compounds **1**–**15** expressed by IC_50_ value varied from 16.4 μmol/L (**3**) to 487.0 μmol/L (**14**). The inhibitory activity of the most active compounds **3** was 8.6 times lower than that of the standard DCMU (IC_50_ = 1.9 μmol/L), a well-known PS2 herbicide. Compounds **6** (R = H), **7** (R = 4-NH_2_) and **13** (R = 2,4-OCH_3_) were inactive and IC_50_ values related to PET inhibition could not be determined. It can be stated that the inhibitory activity increases linearly with increasing lipophilicity of the compounds based on the dependence of PET inhibiting activity of studied dicarbonitriles **1**–**15** on log *P* (Figure 3A) and log *k* (Figure 3B).

**Figure 4:**
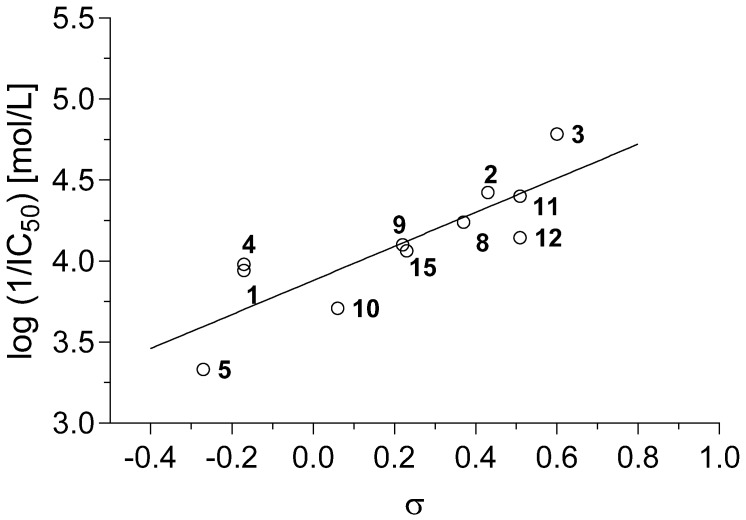
Dependence of PET inhibiting activity of compounds **1**–**15** on the σ constant of the R substituent.

Also if compound **14** (R = 3-NO_2_) was excluded, the dependence of PET inhibiting activity on the Hammett constant σ of R substituent showed again linear dependence (Figure 4). A linear increase of PET-inhibiting activity was observed in the range of σ from -0.15 (**7**; R = 4-NH_2_) to 0.6 (**3**; R = 3,4-Cl).

The correlation between log (1/IC_50_) [mol/L] and log *P* or between log (1/IC_50_) [mol/L] and σ constant of R substituent could be expressed by the following equations:

pI_50_ = 0.577 × (± 0.882) + 1.002 × (± 0.250) log *P* r = 0.950, s = 0.127, F = 82.5, n = 11
(2)

pI_50_ = 3.881 × (± 0.182) + 1.051 × (±0.503) σ
r = 0.844, s = 0.217, F = 22.34, n = 11
(3)

The use of both the above mentioned descriptors (log *P* and σ) in a multilinear correlation did not improve the results of statistical analysis:

pI_50_ = 0.949 × (± 1.669) + 0.887 × (± 0.504) log *P* + 0.160 × (± 0.594) σ
r = 0.952, s = 0.131, F = 38.6, n = 11
(4)

Similarly, better results of statistical analysis were obtained for the correlation between log (1/IC_50_) [mol/L] and log *k* than for multilinear correlation using two descriptors (log *k* and σ).


pI_50_ = 2.703 (±0.414) + 2.835 (±0.816) log *k* r = 0.934, s = 0.144, F = 61.8, n = 11
(5)

pI_50_ = 2.997 × (± 0.331) + 2.034 × (± 0.738) log *k* + 0.481 (0.303) σ
r = 0.976 s = 0.093 F = 80.2 n = 11
(6)

These results indicate that lipophilicity of the compound is determining for PET-inhibiting activity of the studied *N*-substituted 5-amino-6-methylpyrazine-2,3-dicarbonitriles. Lee *et al.* focused on the search for the minimum structural requirements for herbicidal evaluation of 5-(R^1^)-6-(R^2^)-*N*-(R^3^-phenyl)-pyrazine-2-carboxamide analogues as a new class of potent herbicides in a previous paper [31]. Based on IC_50_ values of 19 pyrazine derivatives related to PET inhibition in spinach chloroplasts which were published by Dolezal *et al.* [15], Lee derived and discussed quantitatively 3D-QSARs models between the substituents (R^1^–R^3^) changes of the analogues and their herbicidal activity using comparative molecular field analysis (CoMFA) and comparative molecular similarity indice analysis (CoMSIA) methods. It was predicted that the herbicidal activity increases when large steric substituents were introduced to one part of the *ortho*- and *meta*- positions on the *N*-phenyl ring as R^3^- substituent and small steric substituents on the other part. The same 19 pyrazine derivatives with herbicidal activity were also subjected to the two dimensional quantitative structure activity relationships studies using Vlife Molecular Design 3.0 module which contains various combinations of thermodynamic, electronic, topological and spatial descriptors [32]. It was found that decreasing of number of hydrogen bond acceptor atoms and reduction the any atoms (single, double or triple bonded) separated from any oxygen atom by a seven bond distance in a molecule could be helpful for designing more potent herbicidal agents.

Because the studied *N*-substituted 5-amino-6-methylpyrazine-2,3-dicarbonitriles were found to inhibit the Hill reaction, they can be considered as photosystem 2 (PS2) inhibitors, *i.e.*, PS2 herbicides which ultimately adversely affect growth of weeds as well as agricultural plants. The PS2 inhibitors can act on the donor and/or the acceptor side of PS2. Using EPR spectroscopy it was found that *N*-phenylpyrazine-2-carboxamides interacted with the D^·^ intermediate which is situated at 161^st^ position in D_2_ protein occurring on the donor side of PS2. Due to interaction of these pyrazine derivatives with this part of PS2, the photosynthetic electron transport from the oxygen evolving complex to the reaction centre of PS2 was impaired and consequently, the electron transport between PS2 and PS1 was inhibited [33]. Use of an artificial electron-donor, 1,5-diphenylcarbazide (DPC), acting in the Z^·^/D^·^ intermediate is suitable to estimate whether the studied PET inhibitor acts only on the donor or also on the acceptor side of PS2. Complete restoration of PET after DPC addition to chloroplasts activity of which was inhibited by an inhibitor, indicates that PET between the core of PS2 (P680) and the secondary quinone acceptor Q_B_ was not affected by this inhibitor. Consequently, its site of inhibitory action is situated on the donor side of PS2. However, upon addition of DPC to the studied dicarbonitriles only partial restoration of PET was observed (up to 85% of the control) and therefore it could be assumed that the studied compounds block PET not only by interaction with proteins occurring in the section between the oxygen evolving complex (OEC) and the Z^·^/D^·^ intermediate but to PET inhibition contributes also their interaction with some constituents of photosynthetic apparatus on the acceptor side of PS2. Similar results were obtained previously with 5-*tert*-butyl-*N*-(3-hydroxy-4-chlorophenyl)pyrazine-2-carboxamide and 5-*tert*-butyl-6-chloro-*N-*(3-fluorophenyl)pyrazine-2-carboxamide [33].

**Figure 5:**
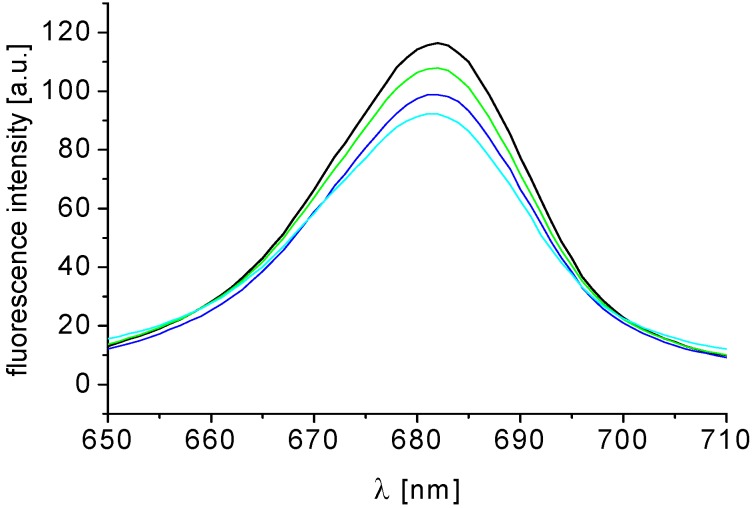
Fluorescence emission spectra of Chl*a* of untreated spinach chloroplasts and chloroplasts treated with 0, 0.13, 0.25 and 0.51 mmol/L of compound **3** (R = 3,4-Cl) (the curves from top to bottom); excitation wave length λ = 436 nm; chlorophyll concentration 10 mg/L.

Compounds **1**–**15** affected the chlorophyll *a* (Chl*a*) fluorescence in spinach chloroplasts. As shown in Figure 5, the intensity of the Chl*a* emission band at 686 nm belonging to the pigment–protein complexes in photosystem 2 decreased in the presence of compound **3** [34]. This finding indicates a perturbation of the Chl*a*–protein complexes in the thylakoid membrane caused by the tested compound. Similar Chl*a* fluorescence decrease in spinach chloroplasts was observed previously with several PET inhibitors, *i.e.*, *N-*benzylpyrazine-2-carboxamides [35], ring-substituted 3-hydroxynaphthalene-2-carboxanilides [36] and 1-hydroxynaphthalene-2-carboxanilides [37], 2-hydroxynaphthalene-1-carboxanilides [38] and ring-substituted 4-arylamino-7-chloroquinolinium chlorides [39].

## 3. Experimental

### 3.1. General

All the chemicals used for preparation of starting compound and final products were purchased from Sigma-Aldrich (Sigma-Aldrich, St. Louis, MO, USA) and were reagent or higher grade of purity. Starting compound was prepared according to proven methodology of conventional organic synthesis. The final aminodehalogenation reactions were performed in CEM Discover microwave reactor with focused field (CEM Corporation, Matthews, NC, USA) connected to the Explorer 24 autosampler (CEM Corporation) and this equipment was running under CEM’s Synergy™ software for monitoring the progress of reactions. The reaction progress was checked by Thin Layer Chromatography (TLC) (Alugram^®^ Sil G/UV254, Machery-Nagel, Postfach, Germany) using 254 nm wavelength UV detection. All the obtained products were purified by crystallization or by preparative flash chromatography (CombiFlash^®^ Rf, Teledyne Isco Inc., Lincoln, NE, USA), using gradient elution with hexane (LacheNer, Neratovice, Czech Republic) and ethyl acetate (Penta, Prague, Czech Republic) as mobile phases. Silica gel (0.040–0.063 nm, Merck, Darmstadt, Germany) was used as the stationary phase. NMR spectra were recorded on Varian Mercury–VxBB 300 (299.95 MHz for ^1^H and 75.43 MHz for ^13^C) or Varian VNMR S500 (499.87 MHz for ^1^H and 125.71 MHz for ^13^C) spectrometers (Varian Corporation, Palo Alto, CA, USA). Chemical shifts were reported in ppm (δ) and were applied indirectly to tetramethylsilane as a signal of solvent (2.49 for ^1^H and 39.7 for ^13^C in DMSO-*d*_6_). Infrared spectra were recorded with spectrometer FT-IR Nicolet 6700 (Thermo Scientific, Waltham, MA, USA) using attenuated total reflectance (ATR) methodology. Melting points were assessed by SMP3 Stuart Scientific (Bibby Scientific Ltd., Staffordshire, UK) and were uncorrected. Elemental analyses were measured with EA 1110 CHNS Analyzer (Fisons Instruments S. p. A., Carlo Erba, Milano, Italy). Calculation of electronic Hammett’s σ parameters was carried out on the software ACD/Percepta ver. 2012 (Advanced Chemistry Development, Inc., Toronto, ON, Canada).

### 3.2. Starting Compound and Final Products Synthesis

The starting compound 5-chloro-6-methylpyrazine-2,3-dicarbonitrile was synthesized in a two-step reaction according to the reported methodology [30]. The first step was a condensation reaction between diaminomaleonitrile (0.025 mol) and pyruvic acid (0.025 mol), which were dissolved in methanol (60 mL), and hydrochloric acid (10 mL, 15%) was added dropwise. It takes two hours to react at room temperature. After the evaporation of two thirds of the solvent, hot water (80 mL) was added and then the rest of methanol was evaporated *in vacuo*. The whole mixture was cooled to 5 °C to initiate the crystallization. The product was collected by suction, dried overnight and subsequently chlorinated with phosphoryl chloride. Product (0.015 mol) was again dissolved in POCl_3_ (0.060 mol) and cooled to 0 °C. Pyridine (0.020 mol) was added dropwise and after the termination of the exothermic reaction, the mixture was heated to 90 °C for 2 h. Excess POCl_3_ was evaporated *in vacuo* and the rest of product was extracted into toluene three or four times, the toluene was evaporated and then the crude product was recrystallized from chloroform. The starting compound (1.12 mmol) was finally treated with 15 variously ring-substituted benzylamines (2.24 mmol) and all these aminodehalogenation reactions took place in the microwave reactor. The conditions used for microwave syntheses were as follows—150 °C, 30 min, 120 W, methanol as a solvent, pyridine as a base and were set experimentally. The reaction was monitored by TLC using hexane/ethyl acetate 2:1 mixture as mobile phase. The final compounds were purified using flash column chromatography with gradient elution using a hexane/ethyl acetate system and if necessary recrystallization from a mixture of ethanol and water.

### 3.3. Analytical Data of the Prepared Compounds

*5-Methyl-6-[(2-methylbenzyl)amino]pyrazine-2,3-dicarbonitrile* (**1**). Light orange crystalline solid. Yield 47.9%; M.p. 152.1–152.9 °C; IR (ATR-Ge, cm^−1^): 3381_m_ (-NH-), 2226_m_ (-CN), 1570_vs_, 1521_s_, 1403_vs_, 1349_m_ (arom.); ^1^H-NMR (500 MHz) δ 8.61 (1H, bs, NH), 7.25–7.10 (4H, m, H3', H4', H5', H6'), 4.56 (2H, s, NCH_2_), 2.47 (3H, s, CH_3_), 2.33 (3H, s, CH_3_); ^13^C-NMR (125 MHz) δ 152.9, 147.5, 136.0, 135.6, 130.2, 130.1, 127.5, 127.2, 125.9, 117.4, 115.7, 114.8, 42.5, 21.3, 19.0; Elemental analysis: calc. for C_15_H_13_N_5_ (MW 263.12): 68.42% C, 4.98% H, 26.60% N; found 68.22% C, 5.17% H, 26.44% N.

*5-Methyl-6-{[3-(trifluoromethyl)benzyl]amino}pyrazine-2,3-dicarbonitrile* (**2**). Yellow-orange crystalline solid. Yield 51.6%; M.p. 158.5–159.7 °C (decomp.); IR (ATR-Ge, cm^−1^): 3377_m_ (-NH-), 2227_m_ (-CN), 1570_vs_, 1521_s_, 1402_vs_, 1353_m_, 1324_vs_ (arom.), 1128_vs_ (-C-F); ^1^H-NMR (300 MHz) δ 8.75 (1H, bs, NH), 7.77–7.60 (1H, m, H2'), 7.68–7.50 (3H, m, H4', H5', H6'), 4.69 (2H, d, *J* = 4.6 Hz, NCH_2_), 2.46 (3H, s, CH_3_); ^13^C-NMR (75 MHz) δ 152.9, 147.7, 139.6, 131.8, 130.0, 129.6, 129.2 (q, *J* = 31.5 Hz), 124.5 (q, *J* = 4.0 Hz), 124.4 (q, *J* = 272.3 Hz), 124.0 (q, *J* = 4.0 Hz), 117.7, 115.6, 114.8, 44.0, 21.2; Elemental analysis: calc. for C_15_H_10_F_3_N_5_ (MW 317.27): 56.78% C, 3.18% H, 22.07% N; found 56.59% C, 3.30% H, 21.93% N.

*5-[(3,4-Dichlorobenzyl)amino]-6-methylpyrazine-2,3-dicarbonitrile* (**3**). Light yellow crystalline solid. Yield 60.9%; M.p. 154.4–156.2 °C; IR (ATR-Ge, cm^−1^): 3329_m_ (-NH-), 2227_m_ (-CN), 1568_vs_, 1516_s_, 1466_m_, 1402_vs_ (arom.); ^1^H-NMR (300 MHz) δ 8.69 (1H, bs, NH), 7.61 (1H, d, *J* = 1.9 Hz, H2'), 7.57 (1H, d, *J* = 8.2 Hz, H5'), 7.33 (1H, dd, *J* = 8.2 Hz, *J* = 1.9 Hz, H6'), 4.59 (2H, s, NCH_2_), 2.46 (3H, s, CH_3_); ^13^C-NMR (75 MHz) δ 152.8, 147.8, 139.4, 131.1, 130.6, 130.0, 129.8, 129.6, 127.9, 117.8, 115.6, 114.8, 43.3, 21.2; Elemental analysis: calc. for C_14_H_9_Cl_2_N_5_ (MW 318.16): 52.85% C, 2.85% H, 22.01% N; found 52.71% C, 3.05% H, 21.88% N.

*5-Methyl-6-[(4-methylbenzyl)amino]pyrazine-2,3-dicarbonitrile* (**4**). Light yellow crystalline solid. Yield 60.5%; M.p. 140.8–142.3 °C; IR (ATR-Ge, cm^−1^): 3370_s_ (-NH-), 2223_m_ (-CN), 1576_vs_, 1519_s_, 1438_m_, 1400_vs_, 1351_s_ (arom.); ^1^H-NMR (300 MHz) δ 8.68 (1H, bs, NH), 7.24–7.18 (2H, m, AA', BB', H2', H6'), 7.14–7.09 (2H, m, AA', BB', H3', H5'), 4.55 (2H, bs, NCH_2_), 2.44 (3H, s, CH_3_), 2.26 (3H, s, CH_3_); ^13^C-NMR (75 MHz) δ 152.8, 147.5, 136.4, 135.0, 130.1, 128.1, 127.5, 117.3, 115.7, 114.9, 44.1, 21.2, 20.8; Elemental analysis: calc. for C_15_H_13_N_5_ (MW 263.30): 68.42% C, 4.98% H, 26.60% N; found 68.30% C, 5.17% H, 26.43% N.

*5-[(4-Methoxybenzyl)amino]-6-methylpyrazine-2,3-dicarbonitrile* (**5**). Ochre crystalline solid. Yield 61.3%; M.p. 153.0–154.2 °C (decomp.); IR (ATR-Ge, cm^−1^): 3345_m_ (-NH-), 2948_w_ (-OCH_3_), 2223_m_ (-CN), 1585_s_, 1570_vs_, 1511_vs_, 1462_m_, 1403_vs_ (arom.); ^1^H-NMR (300 MHz) δ 8.66 (1H, bs, NH), 7.29–7.23 (2H, m, AA', BB', H2', H6'), 6.90–6.84 (2H, m, AA', BB', H3', H5'), 4.52 (2H, bs, NCH_2_), 3.71 (3H, s, OCH_3_), 2.43 (3H, s, CH_3_); ^13^C-NMR (75 MHz) δ 158.6, 152.8, 147.4, 130.1, 129.9, 129.0, 117.3, 115.7, 114.9, 113.9, 55.2, 43.8, 21.2; Elemental analysis: calc. for C_15_H_13_N_5_O (MW 279.30): 64.51% C, 4.69% H, 25.07% N; found 64.35% C, 4.81% H, 24.84% N.

*5-(Benzylamino)-6-methylpyrazine-2,3-dicarbonitrile* (**6**) [30]. Yellow-orange crystalline solid. Yield 53.3%; M.p. 128.7–130.7 °C (described in the literature 118–119 °C); IR (ATR-Ge, cm^−1^): 3404_m_ (‑NH-), 2228_m_ (-CN), 1564_vs_, 1508_vs_, 1454_m_, 1400_vs_, 1357s (arom.); ^1^H-NMR (300 MHz) δ 8.72 (1H, bs, NH), 7.37–7.20 (5H, m, H2', H3', H4', H5', H6'), 4.61 (2H, d, *J* = 3.3 Hz, NCH_2_), 2.45 (3H, s, CH_3_); ^13^C-NMR (75 MHz) δ 152.9, 147.5, 138.1, 130.1, 128.5, 127.5, 127.2, 117.4, 115.7, 114.9, 44.3, 21.2; Elemental analysis: calc. for C_14_H_11_N_5_ (MW 249.27): 67.46% C, 4.45% H, 28.10% N; found 67.21% C, 4.63% H, 27.95% N.

*5-[(4-Aminobenzyl)amino]-6-methylpyrazine-2,3-dicarbonitrile* (**7**). Brown crystalline solid. Yield 37.2%; M.p. 154.9–156.5 °C; IR (ATR-Ge, cm^−1^): 3413_m_ (-NH_2_), 3376_m_ (-NH-), 2234_m_ (-CN), 1572_vs_, 1519_vs_, 1440_m_, 1402_vs_, 1352_s_ (arom.); ^1^H-NMR (300 MHz) δ 8.54 (1H, bs, NH), 7.04–6.94 (2H, m, AA', BB', H2', H6'), 6.54–6.45 (2H, m, AA', BB', H3', H5'), 4.97 (2H, bs, NH_2_), 4.41 (2H, d, *J* = 3.8 Hz, NCH_2_), 2.41 (3H, s, CH_3_); ^13^C-NMR (75 MHz) δ 152.7, 148.0, 147.3, 130.2, 128.7, 124.7, 117.0, 115.8, 114.9, 113.9, 44.2, 21.2; Elemental analysis: calc. for C_14_H_12_N_6_ (MW 264.29): 63.62% C, 4.58% H, 31.80% N; found 63.48% C, 4.45% H, 31.65% N.

*5-[(3-Chlorobenzyl)amino]-6-methylpyrazine-2,3-dicarbonitrile* (**8**). Yellow crystalline solid. Yield 60.2%; M.p. 151.0–151.8 °C; IR (ATR-Ge, cm^−1^): 3306_m_ (-NH-), 2248_m_ (-CN), 1567_vs_, 1513_vs_, 1468_m_, 1401_vs_, 1351_s_ (arom.); ^1^H-NMR (300 MHz) δ 8.70 (1H, t, *J* = 5.9 Hz, NH), 7.43–7.39 (1H, m, H2'), 7.38–7.27 (3H, m, H4', H5', H6'), 4.61 (2H, d, *J* = 5.9 Hz, NCH_2_), 2.46 (3H, s, CH_3_); ^13^C-NMR (75 MHz) δ 152.9, 147.7, 140.7, 133.2, 130.4, 130.0, 127.4, 127.2, 126.2, 117.7, 115.6, 114.9, 43.8, 21.2; Elemental analysis: calc. for C_14_H_10_ClN_5_ (MW 283.72): 59.27% C, 3.55% H, 24.68% N; found 59.08% C, 3.71% H, 24.57% N.

*5-[(2-Chlorobenzyl)amino]-6-methylpyrazine-2,3-dicarbonitrile* (**9**). Dark orange crystalline solid. Yield 24.7%; M.p. decomp.; IR (ATR-Ge, cm^−1^): 3381_m_ (-NH-), 2227_m_ (-CN), 1570_vs_, 1521_s_, 1474_m_, 1440_m_, 1401_s_, 1351_s_ (arom.); ^1^H-NMR (300 MHz) δ 8.68 (1H, bs, NH), 7.51–7.24 (4H, m, H3', H4', H5', H6'), 4.66 (2H, s, NCH_2_), 2.49 (3H, s, CH_3_); ^13^C-NMR (75 MHz) δ 153.0, 147.7, 134.9, 132.3, 130.0, 129.4, 129.1, 128.9, 127.4, 117.8, 115.6, 114.8, 42.4, 21.2; Elemental analysis: calc. for C_14_H_10_ClN_5_ (MW 283.72): 59.27% C, 3.55% H, 24.68% N; found 59.08% C, 3.68% H, 24.90% N.

*5-[(2-Fluorobenzyl)amino]-6-methylpyrazine-2,3-dicarbonitrile* (**10**). Dark yellow crystalline solid. Yield 60.8%; M.p. 134.8–136.3 °C; IR (ATR-Ge, cm^−1^): 3377_m_ (-NH-), 2231_m_ (-CN), 1573_vs_, 1523_s_, 1491_m_, 1402_s_, 1354_s_ (arom.); ^1^H-NMR (300 MHz) δ 8.68 (1H, t, *J* = 5.3 Hz, NH), 7.42–7.10 (4H, m, H3', H4', H5', H6'), 4.63 (2H, d, *J* = 5.3 Hz, NCH_2_), 2.46 (3H, s, CH_3_); ^13^C-NMR (75 MHz) δ 160.0 (d, *J* = 244.8 Hz), 152.9, 147.6, 129.8 (d, *J* = 20.4 Hz), 129.8, 129.4 (d, *J* = 8.3 Hz), 124.6 (d, *J* = 17.8 Hz), 124.5 (d, *J* = 6.6 Hz), 117.7, 115.5 (d, *J* = 9.2 Hz), 115.2, 114.8, 38.3, 21.2; Elemental analysis: calc. for C_14_H_10_FN_5_ (MW 267.26): 62.92% C, 3.77% H, 26.20% N; found 62.71% C, 3.59% H, 26.38% N.

*5-Methyl-6-{[4-(trifluoromethyl)benzyl]amino}pyrazine-2,3-dicarbonitrile* (**11**). Light yellow crystalline solid. Yield 38.3%; M.p. 148.4–149.6 °C; IR (ATR-Ge, cm^−1^): 3397_m_ (-NH-), 2232_m_ (-CN), 1570_vs_, 1520_s_, 1422_m_, 1399_s_, 1324_vs_ (arom.), 1114_vs_ (-C-F); ^1^H-NMR (300 MHz) δ 8.54 (1H, t, *J* = 5.3 Hz, NH), 7.71–7.64 (2H, m, AA', BB', H2', H6'), 7.38–7.52 (2H, m, AA', BB', H3', H5'), 4.69 (2H, d, *J* = 5.3 Hz, NCH_2_), 2.51 (3H, s, CH_3_); ^13^C-NMR (75 MHz) δ 152.9, 147.7, 143.1, 130.0, 128.2, 127.9 (q, *J* = 31.5 Hz), 125.4 (q, *J* = 3.7 Hz), 124.5 (q, *J* = 272.0 Hz), 117.8, 115.6, 114.8, 44.0, 21.2; Elemental analysis: calc. for C_15_H_10_F_3_N_5_ (MW 317.27): 56.78% C, 3.18% H, 22.07% N; found 56.59% C, 2.99% H, 21.91% N.

*5-Methyl-6-{[2-(trifluoromethyl)benzyl]amino}pyrazine-2,3-dicarbonitrile* (**12**). Yellow-orange crystalline solid. Yield 49.1%; M.p. 157.8–159.4 °C (decomp.); IR (ATR-Ge, cm^−1^): 3408_m_ (-NH-), 2230_m_ (-CN), 1569_vs_, 1522_s_, 1430_m_, 1409_s_, 1398_s_, 1368_s_, 1314_vs_ (arom.), 1114_vs_ (-C-F); ^1^H-NMR (300 MHz) δ 8.76 (1H, bs, NH), 7.77–7.71 (1H, m, H3'), 7.64–7.43 (3H, m, H4', H5', H6'), 4.77 (2H, bs, NCH_2_), 2.51 (3H, s, CH_3_); ^13^C-NMR (75 MHz) δ 153.0, 147.7, 136.3, 132.9, 130.0, 128.3, 127.8, 126.4 (q, *J* = 30.3 Hz), 126.1 (q, *J* = 6.0 Hz), 124.7 (q, *J* = 274.0 Hz), 118.0, 115.5, 114.7, 41.1, 21.2; Elemental analysis: calc. for C_15_H_10_F_3_N_5_ (MW 317.27): 56.78% C, 3.18% H, 22.07% N; found 56.62% C, 3.08% H, 22.00% N.

*5-[(2,4-Dimethoxybenzyl)amino]-6-methylpyrazine-2,3-dicarbonitrile* (**13**). Ochre crystalline solid. Yield 62.3%; M.p. 151.6–152.9 °C; IR (ATR-Ge, cm^−1^): 3336_m_ (-NH-), 2924_w_ (-OCH_3_), 2224_m_ (-CN), 1609_s_, 1574_vs_, 1509_vs_, 1467_m_, 1440_s_, 1405_vs_ (arom.); ^1^H-NMR (300 MHz) δ 8.45 (1H, t, *J* = 5.7 Hz, NH), 7.07 (1H, d, *J* = 8.2 Hz, H6'), 6.55 (1H, d, *J* = 2.5 Hz, H3'), 6.43 (1H, dd, *J* = 8.2 Hz, *J* = 2.5 Hz, H5'), 4.47 (2H, d, *J* = 5.7 Hz, NCH_2_), 3.80 (3H, s, OCH_3_), 3.72 (3H, s, OCH_3_), 2.44 (3H, s, CH_3_); ^13^C-NMR (75 MHz) δ 160.1, 158.1, 153.0, 147.4, 130.2, 128.8, 117.3, 117.2, 115.8, 114.9, 104.6, 98.5, 55.7, 55.4, 39.5, 21.3; Elemental analysis: calc. for C_16_H_15_N_5_O_2_ (MW 309.32): 62.13% C, 4.89% H, 22.64% N; found 62.36% C, 5.01% H, 22.52% N.

*5-Methyl-6-[(3-nitrobenzyl)amino]pyrazine-2,3-dicarbonitrile* (**14**). Orange-red crystalline solid. Yield 17.2%; M.p. decomp.; IR (ATR-Ge, cm^−1^): 3394_m_ (-NH-), 2231_m_ (-CN), 1571_vs_, 1547_m_, 1524_vs_, 1478_m_, 1430_m_, 1396_s_, 1366_s_, 1348_vs_ (arom.); ^1^H-NMR (300 MHz) δ 8.79 (1H, t, *J* = 5.8 Hz, NH), 8.23 (1H, s, H2'), 8.11 (1H, d, *J* = 7.8 Hz, H4'), 7.81 (1H, d, *J* = 7.8 Hz, H6'), 7.62 (1H, t, *J* = 7.8 Hz, H5'), 4.73 (2H, d, *J* = 5.8 Hz, NCH_2_), 2.47 (3H, s, CH_3_); ^13^C-NMR (75 MHz) δ 152.9, 148.0, 147.8, 140.6, 134.4, 130.0, 130.0, 122.5, 122.3, 117.9, 115.6, 114.8, 43.8, 21.2; Elemental analysis: calc. for C_14_H_10_N_6_O_2_ (MW 294.27): 57.14% C, 3.43% H, 28.56% N; found 56.93% C, 3.64% H, 28.56% N.

*5-[(4-Chlorobenzyl)amino]-6-methylpyrazine-2,3-dicarbonitrile* (**15**). Brown crystalline solid. Yield 30.8%; M.p. 152.8–154.8 °C (decomp.); IR (ATR-Ge, cm^−1^): 3378_m_ (-NH-), 2230_m_ (-CN), 1570_vs_, 1521_s_, 1487_m_, 1441_m_, 1403_s_, 1347_s_ (arom.), 804_s_ (-C-Cl); ^1^H-NMR (500 MHz) δ 8.42 (1H, bs, NH), 7.39–7.33 (4H, m, H2', H3', H5', H6'), 4.59 (2H, s, NCH_2_), 2.45 (3H, s, CH_3_); ^13^C-NMR (125 MHz) δ 152.8, 147.6, 137.2, 131.8, 130.0, 129.4, 128.5, 117.6, 115.6, 114.8, 43.7, 21.2; Elemental analysis: calc. for C_14_H_10_ClN_5_ (MW 283.72): 59.27% C, 3.55% H, 24.68% N; found 59.16% C, 3.65% H, 24.62% N.

### 3.4. Lipophilicity HPLC Determination and Calculations

Experimental lipophilicity parameter log *k* was ascertained using an Agilent Technologies 1200 SL HPLC system with a SL G1315C Diode-Array Detector, ZORBAX XDB-C18 5 μm, 4 × 4 mm, Part No. 7995118-504 chromatographic pre-column and ZORBAX Eclipse XDB-C18 5 μm, 4.6 × 250 mm, Part No. 7995118-585 column (Agilent Technologies Inc., Colorado Springs, CO, USA). The separation process was controlled by Agilent ChemStation, version B.04.02 extended by spectral module (Agilent Technologies Inc.). A solution of MeOH (HPLC grade, 70%) and H_2_O (HPLC-Milli-Q Grade, 30%) was used as mobile phase. The total flow of the column was 1.0 mL/min, injection 20 μL, column temperature 30 °C. Detection wavelength λ= 210 nm and monitor wavelength λ= 270 nm were chosen for this measurement. The KI methanol solution was used for the dead time (TD) determination. Retention times (TR) of synthesized compounds were measured in minutes. The capacity factors *k* were calculated using Microsoft Excel according to formula *k* = (TR − TD)/TD, where TR is the retention time of the solute and TD denotes the dead time obtained *via* an unretained analyte. Log *k*, calculated from the capacity factor *k*, is used as the lipophilicity index converted to log *P* scale. Values of log *P* and Clog *P* were calculated with the PC programme CS ChemBioDraw Ultra 13.0 (CambridgeSoft, Cambridge, MA, USA).

### 3.5. Biological Assays

#### 3.5.1. Antimycobacterial *In Vitro* Screening

Mycobacterial screening was performed against four mycobacterial stems (*M. tuberculosis* H37Rv CNCTC My 331/88, *M. kansasii* CNCTC My 235/80, *M. avium ssp. avium* CNCTC My 80/72, *M. avium* CNCTC My 152/73 (Czech National Collection of Type Cultures, National Institute of Public Health, Prague, Czech Republic)) using isoniazid and pyrazinamide (Sigma-Aldrich) as standards. Culturing medium was Sula’s semisynthetic medium (Trios, Prague, Czech Republic) with pH 6.0. Tested compounds were dissolved in dimethylsulfoxide (DMSO) and diluted with medium to final concentrations 100, 50, 25, 12.5, 6.25, 3.125 and 1.5625 μg/mL. The method used for this assay was microdilution broth panel method. The final concentration of DMSO did not exceed 1% (v/v) and did not affect the growth of *Mycobacteria*. The cultures were grown in Sula’s medium at 37 °C in humid dark atmosphere. The antimycobacterial activity was determined using Alamar Blue colouring after 14 days, resp. 6 days against *M. kansasii,* of incubation as MIC (µg/mL). This evaluation was done in cooperation with Department of Clinical Microbiology, University Hospital in Hradec Kralove, Hradec Kralove, Czech Republic.

#### 3.5.2. Antimycobacterial *In Vitro* Screening Against *M. smegmatis*

This assay was focused on the activity against fast growing *Mycobacterium smegmatis* MC2155 (CIT Collection, Cork Institute of Technology, Cork, Ireland). The method used there was also microdilution broth panel method and as the medium was used Middlebrook 7H9 Broth with 10% of OADC supplement (Sigma-Aldrich). Tested compounds were dissolved in DMSO and medium and the final concentrations were set as 1000, 500, 250, 125, 62.5, 31.25, 15.625 and 7.8125 µg/mL. The final concentration of DMSO did not exceed 2% (v/v) and did not affect the growth of *M. smegmatis*. The standards used for determination of activity were isoniazid and ciprofloxacin. MIC was read after 48 h of incubation at 37 °C, addition of Alamar Blue stain was followed by 12 h of additional incubation with this reagent. This screening was performed under the patronage of the Department of Biological Sciences, Cork Institute of Technology, Cork, Ireland.

#### 3.5.3. Antifungal and Antibacterial *In Vitro* Screenings

Antibacterial evaluation was made using the microdilution broth method in plates M27A-M1 (200+10) against eight bacterial stems from the Czech Collection of Microorganisms (Brno, Czech Republic) or clinical isolates from Department of Clinical Microbiology, University Hospital in Hradec Kralove (Hradec Kralove, Czech Republic) (*Staphylococcus aureus* CCM 4516/08, *Staphylococcus aureus* H 5996/08 methicillin resistant, *Staphylococcus epidermidis* H 6966/08, *Enterococcus* sp. J14365/08, *Escherichia coli* CCM 4517, *Klebsiella pneumoniae* D 11750/08, *Klebsiella pneumoniae* J 14368/05 ESBL positive, *Pseudomonas aeruginosa* CCM 1961). Mueller Hinton broth was used for the cultivation that was done in humid atmosphere at 35 °C. The readings were made after 24 and 48 h and MIC was set as 80% inhibition of control. The standards were neomycin, bacitracin, penicillin G, ciprofloxacin and phenoxymethylpenicillin and tested products were dissolved in DMSO (final concentration of DMSO did not exceed 1% (v/v)) [40].

Antifungal evaluation was also accomplished with microdilution broth method. On the contrary, there was used RPMI 1640 broth with glutamine as medium and conditions were humid and dark atmosphere, pH 7.0 (buffered with 3-morpholinopropane-1-sulfonic acid) and 35 °C. Eight fungal strains were used (*Candida albicans* ATCC 44859, *Candida tropicalis* 156, *Candida krusei* E28, *Candida glabrata* 20/I, *Trichosporon asahii* 1188, *Aspergillus fumigatus* 231, *Absidia corymbifera* 272, *Trichophyton mentagrophytes* 445) together with 4 antimycotic standards amphotericin B, voriconazole, nystatin and fluconazole. The MIC was set as 80% inhibition of control and readings were made after 24 and 48 h (50% IC, 72 and 120 h for fibrous fungi) and tested compounds were also dissolved in DMSO (final concentration of DMSO in medium did not exceed 2.5% (v/v)) [41].

#### 3.5.4. Study of the Inhibition of Oxygen Evolution Rate in Spinach Chloroplasts

Chloroplasts were prepared from spinach (*Spinacia oleracea* L.) according to Masarovicova and Kralova [42]. The inhibition of photosynthetic electron transport (PET) in spinach chloroplasts was determined spectrophotometrically (Genesys 6, Thermo Scientific, Madison, WI, USA) using an artificial electron acceptor 2,6-dichlorophenol-indophenol (DCPIP) according to Kralova *et al.* [43] and the rate of photosynthetic electron transport (PET) was monitored as a photo-reduction of DCPIP. The measurements were carried out in a phosphate buffer (0.02 mol/L, pH 7.2) containing sucrose (0.4 mol/L), MgCl_2_ (0.005 mol/L) and NaCl (0.015 mol/L). The chlorophyll content was 30 mg/L in these experiments and the samples were irradiated (~100 W/m^2^) from a 10 cm distance with halogen lamp (250 W) using a 4 cm water filter to prevent warming of the samples (suspension temperature 4 °C). The studied compounds were dissolved in DMSO due to their limited water solubility. The applied DMSO concentration (up to 4% (v/v)) did not affect the photochemical activity in spinach chloroplasts (PET). The inhibitory efficiency of the studied compounds was expressed as the IC_50_ values, *i.e.*, molar concentration of the compounds causing 50% decrease in the oxygen evolution relative to the untreated control. The comparable IC_50_ value for a selective herbicide 3-(3,4-dichlorophenyl)-1,1-dimethylurea (Diurone^®^, DCMU) was about 1.9 μmol/L [44].

#### 3.5.5. Study of Fluorescence of Chlorophyll *a* in Spinach Chloroplasts

The fluorescence emission spectra of chlorophyll *a* (Chl*a*) in spinach chloroplasts were recorded on fluorescence spectrophotometer F-2000 (Hitachi, Tokyo, Japan) using excitation wavelength λ_ex_ = 436 nm for monitoring fluorescence of Chl*a*, excitation slit 20 nm and emission slit 10 nm. The samples were kept in the dark 2 min before measuring. The phosphate buffer used for dilution of the chloroplast suspension was the same as described above. Due to low aqueous solubility the compounds were added to a chloroplast suspension in DMSO solution. The DMSO concentration in all samples was the same as in the control (10% (v/v)). The chlorophyll concentration in chloroplast suspension was 10 mg/L.

## 4. Conclusions

A series of 15 pyrazinamide derivatives (14 of them novel) was synthesized by aminodehalogenation reactions focusing on microwave assisted synthesis. All the final compounds were characterized with IR, NMR and other analytical data and then subjected to *in vitro* evaluation in order to discover their potential antimycobacterial, antifungal, antibacterial and herbicidal activities.

The lipophilicity was measured using RP-HPLC methodology and also calculated or predicted with the PC program CS ChemBioDraw Ultra 13.0. These values were compared and the dependence between log *k* and log *P* was linear.

In antimycobacterial screening compounds **3** (R = 3,4-Cl), **9** (R = 2-Cl) and **11** (R = 4-CF_3_) showed good activity against wild strain *M. tuberculosis* H37Rv (MIC = 6.25 µg/mL) compared to the standards pyrazinamide (MIC = 12.5 µg/mL) and isoniazid (MIC = 1.56 µg/mL). Compounds **11** (R = 4-CF_3_) and **12** (R = 2-CF_3_) were active against the non-tuberculosis strains *M. kansasii* and *M. avium* as well. Although the majority of synthesized compounds were active, there is no clear dependence between lipophilicity and antimycobacterial activity, but it can be stated that the most potent substances were also from the group of most lipophilic compounds and the most favourable substitutions are the electron-withdrawing groups such as chlorine or trifluoromethyl. Activity against fast growing *Mycobacteria* was also determined but no active substances were identified.

No interesting results were observed in antibacterial and antifungal screenings. Three compounds (**7** (R = 4-NH_2_), **10** (R = 2-F), and **15** (R = 4-Cl)) showed insignificant activity against the fungus *Trichophyton mentagrophytes*, which was found to be worse compared to the standards. The rest of substances showed no *in vitro* antibacterial or antifungal activity.

On the contrary, *N*-substituted 5-amino-6-methylpyrazine-2,3-dicarbonitriles were found to inhibit the Hill reaction in spinach chloroplasts which indicated that these compounds act as PS2 inhibitors.The IC_50_ values related to PET inhibition varied in the investigated set in the range from 16.4 μmol/L (**3**; R = 3,4-Cl) to 487.0 μmol/L (**14**; R = 3-NO_2_). The lipophilicity of the compounds was determinant for PET‑inhibiting activity. The site of inhibitory action of studied compounds in the photosynthetic apparatus is situated both on the donor and on the acceptor side of PS2. Perturbation of the Chl*a*-protein complexes in the thylakoid membranes caused by the tested compounds was documented by a decrease of the Chl*a* emission band intensity at 686 nm belonging to the pigment‑protein complexes in photosystem 2.

Based on the obtained results it can be assumed that the antifungal and antibacterial activities of studied compounds are not directly dependent on lipophilicity. This conclusion cannot be applied for the herbicidal activity because there is a linear dependence between activity and lipophilicity. Dependence between antimycobacterial activity and the benzyl substituents was found. This reliance was expressed by the lipophilicity parameter log *k* resp. π constant and showed the importance of the ring-substituted benzyl moiety.

## Supplementary Materials

Supplementary File 1
