# Supplementary material for: N-Substituted 5-Amino-6-methylpyrazine-2,3-dicarbonitriles: Microwave-Assisted Synthesis and Biological Properties
Source: Molecules. 2014 Jan 7;19(1):651–71. doi: 10.3390/molecules19010651 (PMC6270751; doi:10.3390/molecules19010651)
Supplement: Supplementary File 1 [file molecules-19-00651-s001.pdf]

Correction of Acknowledgments

The publication is co-financed by the European Social Fund and the state budget of the Czech Republic. Project No. CZ.1.07/2.3.00/20.0235, the title of the project: TEAB. This study was also supported by the Ministry of Health of Czech Republic (IGA NZ 13346), Grant Agency of Charles University B-CH/710312 and SVV 260 062 as well as by the Slovak Grant Agency VEGA, Grant No. 1/0612/11, by the Project APVV-0061-11 and by Sanofi-Aventis Pharma Slovakia. The authors also wish to thank Ida Dufkova for performing and evaluating the antifungal and antibacterial assays and Barbora Servusova and Jan Zitko for English revisions.
